# Longitudinal stability of cortical grey matter measures varies across brain regions, imaging metrics, and testing sites in the ABCD study

**DOI:** 10.1162/imag_a_00086

**Published:** 2024-03-19

**Authors:** Sam Parsons, Andreas M. Brandmaier, Ulman Lindenberger, Rogier Kievit

**Affiliations:** Donders Institute for Brain, Cognition and Behavior, Radboud University Medical Center, Nijmegen, Netherlands; Center for Lifespan Psychology, Max Planck Institute for Human Development, Berlin, Germany; Department of Psychology, MSB Medical School Berlin, Berlin, Germany; Max Planck UCL Centre for Computational Psychiatry and Ageing Research, Berlin, Germany; Medical Research Council Cognition and Brain Sciences Unit, University of Cambridge, Cambridge, United Kingdom

**Keywords:** longitudinal stability, ABCD, developmental neuroscience, structural MRI, intraclass effect decomposition

## Abstract

Magnetic resonance imaging (MRI) is a vital tool for the study of brain structure and function. It is increasingly being used in individual differences research to examine brain-behaviour associations. Prior work has demonstrated low test-retest stability of functional MRI measures, highlighting the need to examine the longitudinal stability (test-retest reliability across long timespans) of MRI measures across brain regions and imaging metrics, particularly in adolescence. In this study, we examined the longitudinal stability of grey matter measures (cortical thickness, surface area, and volume) across brain regions, and testing sites in the Adolescent Brain Cognitive Development (ABCD) study release v4.0. Longitudinal stability ICC estimates ranged from 0 to .98, depending on the measure, parcellation, and brain region. We used Intra-Class Effect Decomposition (ICED) to estimate between-subjects variance and error variance, and assess the relative contribution of each across brain regions and testing sites on longitudinal stability. In further exploratory analyses, we examined the influence of parcellation used (Desikan-Killiany-Tourville and Destrieux) on longitudinal stability. Our results highlight meaningful heterogeneity in longitudinal stability across brain regions, structural measures (cortical thickness in particular), parcellations, and ABCD testing sites. Differences in longitudinal stability across brain regions were largely driven by between-subjects variance, whereas differences in longitudinal stability across testing sites were largely driven by differences in error variance. We argue that investigations such as this are essential to capture patterns of longitudinal stability heterogeneity that would otherwise go undiagnosed. Such improved understanding allows the field to more accurately interpret results, compare effect sizes, and plan more powerful studies.

## 1 Introduction

Brain imaging techniques, including Magnetic Resonance Imaging (MRI), are indispensable for studying brain function and structure and its role in supporting cognitive development across the lifespan. In recent years, MRI has been increasingly used to examine individual differences, suggesting that, for instance, individuals with (regional) differences in cortical morphology or structural connectivity also demonstrate differences in phenotypes such as cognitive performance (Kievit et al., 2014; Magistro et al., 2015; Muetzel et al., 2015; Schnack et al., 2015). Moreover, the crucial role of (differences in) change and maturation in brain structure across the lifespan has prompted longitudinal investigations collecting multiple brain scans from individuals across the lifespan (e.g., Casey et al., 2018; Healthy Brain Study Consortium et al., 2021; von Rhein et al., 2015; Walhovd et al., 2018). Addressing individual differences questions, whether cross-sectionally or longitudinally, rests on the assumption that brain imaging measures are reliable. In other words, the inferences we can draw from such longitudinal datapoints depend on the extent to which they capture stable between-subjects differences with little contamination by within-subject fluctuations or measurement error.

More commonly than not, we do not know how reliable our measures are (Flake et al., 2017; Gawronski et al., 2011; Hussey & Hughes, 2018; Parsons et al., 2019). This basic psychometric concern does not only relate to questionnaires, but also cognitive measurements (Parsons et al., 2019) and neuroimaging metrics (Anand et al., 2022; Brandmaier, Wenger, et al., 2018; Noble et al., 2017; Wenger et al., 2021; Zuo et al., 2019). Low reliability translates to low statistical power and related challenges, including a decreased likelihood that a significant finding reflects a true effect (Button et al., 2013), is in the correct direction (Type 2 “Sign” error; Gelman & Carlin, 2014), and an inherent overestimation of the true effect size (Type M “Magnitude” error; Gelman & Carlin, 2014). In short, if reliability is not assessed, it is impossible to gauge its impact on our results and therefore the confidence we should have them. Failing to assess reliability can become a greater, more complex, problem when we wish to compare effect sizes from different regions, measures, or studies (e.g., see Cooper et al., 2017). For example, a study may conclude that there is no difference in brain atrophy between an experimental medicine group and a control group, when in fact the clinical benefits are attenuated or hidden because of low reliability. Similarly, within studies, marked differences in reliability between brain regions could lead researchers to make incorrect conclusions about the similarity of brain-behaviour associations across these regions. As such, we propose that mapping reliability across brain regions and measures provides vital information about reliability heterogeneity. Further, exploring reliability heterogeneity may allow us to uncover sources of unreliability, and account for this in our study designs to improve precision, statistical power, and efficiency (Brandmaier et al., 2015; Brandmaier, Wenger, et al., 2018; Noble et al., 2017; Zuo et al., 2019).

### 1.1 Reliability and stability

Consider two brain scans collected from the same individual. If, hypothetically, we observe no differences between brain images, we can infer our measure is perfectly reliable. If the second scan was obtained immediately following the first, we can assume that any differences between scans are due to some measurement error introduced during the scans or image processing, and that greater differences between these scans indicate lower reliability. However, as the time between scans increases, the difference between successive scans will reflect a combination of (un)reliability as well as true differences, or changes, in brain structure. For example, time of day (Karch et al., 2019) and hydration levels (e.g., Trefler et al., 2016) may induce differences between scans. When scans are taken months or years apart (Casey et al., 2018; Kennedy et al., 2022), it is highly likely that developmental processes have occurred: brain structure changes over time, and the rate of change depends on the region, imaging modality, and lifespan stage (Bethlehem et al., 2022). Moreover, impactful events that occur in between scans such as learning new skills (e.g., Wenger et al., 2021), or adverse events such as brain injury (e.g., Lindberg et al., 2019), will lead to lasting differences. As such, differences in brain images over years necessarily reflect a combination of measurement reliability and longitudinal stability.

Traditional models used to estimate reliability focus on measurement properties and thus implicitly assume stability, that is, no systematic changes or individual differences in change over time (Nesselroade, 1991). When we use these models to estimate reliability over long durations, individual differences in change will appear as error in our model. To address this challenge, prior work tracing back to Cronbach and Furby (1970; also see Hertzog & Nesselroade, 2003) has denoted reliability estimates from these models as *stability* (Brandmaier, Wenger, et al., 2018; Deary et al., 2013; Kennedy et al., 2022). The difference in interpretation relies on the tenability of the assumption that true change in the underlying system is negligible for the purposes of our repeated measurements or not. To reflect this inherent ambiguity, we follow previous work and use the term **longitudinal stability** to describe what is captured by our estimates. At the same time, we emphasise that our estimates capture a mixture of *both reliability and stability* due to expected individual differences in changes in brain structure over the lifespan. With appropriate study designs, it will be possible to disentangle these distinct sources of variance, but the vast majority of longitudinal designs do not (yet) allow for this—an issue we consider further in the discussion. With the emergence of developmental or lifespan studies with long inter-scan intervals (several years in some studies), it is crucial that methodological work allows us to characterise the distinct sources of longitudinal stability across developmental time.

### 1.2 Reliability in brain imaging

Various tools exist to examine reliability. Readers may commonly see Cronbach’s alpha reported to index the internal-consistency reliability of questionnaires (though alternatives like MacDonalds Omega are likely more suitable; McNeish, 2018). Readers may also commonly see a Pearson correlation, or better an Intraclass Correlation Coefficient (Koo & Li, 2016), to index the test-retest reliability of a measure. Broadly, the ICC quantifies the proportion of variance attributed to between-subjects variance compared to all sources of variance (including not only error, but also within-participant variance including between-sessions variance). Due to these strengths, ICCs are becoming more commonly reported in brain imaging (Noble et al., 2021). Various extensions and generalisations of the ICC exist which focus on distinct aspects such as the reliability of a single measurement, or the average of more than one, and whether one wishes to capture absolute agreement or consistency across repeated measures (for a complete introduction, see Koo & Li, 2016).

Although empirical investigations into the (un)reliability of (f)MRI are somewhat limited, existing evidence strongly suggests this reliability is considerably worse than hoped. For instance, analyses of the Adolescent Brain Cognitive Development study (ABCD; Casey et al., 2018) data showed very poor within-session reliability and 2-year stability, of task-based fMRI measures, with estimates (proportion of non-scanner related between-subjects variance to all sources of variance) rarely exceeding .2 (Kennedy et al., 2022). One review of reported test-retest estimates (ICC) also found fMRI measures to have low reliability (mean ICC = .44; Bennett & Miller, 2010) and concluded that studies are needed that examine the factors that influence reliability. In Bennett and Miller, study test-retest intervals varied from less than 1 hour to 59 weeks, and the authors highlight a trend for lower reliability (stability) in studies with test-retest intervals longer than 3 months, relative to studies with intervals less than 1 hour. A recent meta-analysis including 90 experiments using common fMRI tasks found ICC to be around .4 (Elliott et al., 2020). Test-retest intervals varied from 1 day to 1,008 days; however, unlike Bennett & Miller, the authors found no moderating effect of test-retest interval on the meta-analytic ICC estimate. The authors identified various design factors, including scanner, subject, task, and study factors, which may help improve test-retest reliability of fMRI measures in studies of development. It is likely these recommendations are also applicable to structural MRI. Two related considerations are the size of the contribution to reliability and how difficult it is to modify (e.g., adapting study design, increasing the number of scans, etc). For example, Karch and colleagues found increased time between scans and scanning at inconsistent times of day (within and between participants) predicted reliability of several brain volume estimates (Karch et al., 2019). Maintaining the same scanning time for a participant should be a relatively easy way to boost reliability by a small increment. In contrast, additional scanning sessions quickly increase the time and cost of a study.

There is some evidence that structural measures (e.g., cortical thickness) are more reliable than functional measures (Elliott et al., 2020; Han et al., 2006). For example, in one of the few studies to examine test-retest reliability of structural measures, Elliott et al. (2020) analysed data from the Human Connectome Project (HPC; participants aged 25-35, mean time between scans 140 days) and the Dunedin Multidisciplinary Health and Development Study (participants aged 45, mean time between scans 79 days). Across brain regions, cortical thickness ICCs ranged from .547 to .964 in the HPC and .385 to .975 in the Dunedin study, surface area ICCs ranged from .526 to .992 in the HPC and .572 to .991 in the Dunedin study. These results highlight meaningful variation in reliability across brain measures and brain regions. Further, is it reason to suspect that the influence data processing decisions (Li et al., 2021; Parsons, 2022), including parcellation (Mikhael & Pernet, 2019; Yaakub et al., 2020), have on the data also leads to differences in the longitudinal stability of those data? If left undiagnosed, this reliability heterogeneity can have impactful downstream consequences on the inferences we can draw from brain imaging research.

### 1.3 Generating detailed maps of test-retest stability

In this study, we make use of the Adolescent Brain Cognitive Development longitudinal study imaging data (ABCD; Casey et al., 2018; Compton et al., 2019; https://abcdstudy.org/) to map longitudinal stability of structural brain imaging measures. The ABCD study is a collaboration across 21 research sites across the United States, including a representative sample of over 11,000 children aged 9-10, with plans to follow-up participants into young adulthood. For our purposes, the data include two brain imaging sessions at baseline and 2-year follow-up. Relative to prior investigations of structural and functional MRI longitudinal stability, ABCD also offers a considerably larger sample size. For example, the estimates reported by Elliott et al. (2020) from the large-scale Human Connectome Project (Van Essen et al., 2013) and Dunedin study (Poulton et al., 2015), included only 45 and 20 participants with repeated measures, respectively. Further, with the ABCD data we had a decent test-retest sample size for each site (minimum site n = 336), allowing us to isolate these sources of (un)reliability, giving us confidence in the precision of our multigroup analyses across testing sites.

In addition, we note that the opportunities to examine brain-behaviour associations using the ABCD data are vast (Feldstein Ewing et al., 2018). Hundreds of studies using the ABCD data have already been published. Given this, there is increasing importance to generate maps of longitudinal stability specifically for this cohort, to inform data users about potential undiagnosed heterogeneity in longitudinal stability. We had two main questions. First, what is the longitudinal stability of grey matter measures in the ABCD study, and do they differ across brain regions, structural metrics, and testing sites? Second, are these differences in longitudinal stability driven more by individual differences or measurement error?

## 2 Methods

### 2.1 ABCD data

We used imaging data from the Adolescent Brain Cognitive Development study (Casey et al., 2018), data release 4.0 (http://dx.doi.org/10.15154/1523041; see Supplementary Materials for full acknowledgement). Full design information about the ABCD study has been described previously, including: recruitment and sampling procedures (Compton et al., 2019), imaging protocol (Casey et al., 2018), details of image processing (Hagler et al., 2019), guides for researchers using this data (Saragosa-Harris et al., 2022), and open access data from an adult equivalent of ABCD with an accelerated design (Rapuano et al., 2022).

#### 2.1.1 MRI imaging

The raw imaging data were processed using FreeSurfer, version 5.3.0 (Fischl, 2012; Laboratory for Computational Neuroimaging, n.d.) by the ABCD Data Acquisition and Integration Core with a standardised ABCD pipeline (Hagler et al., 2019). Participants’ images were excluded if severe imaging artifacts were detected in manual quality control checks. The Desikan-Killiany-Tourville atlas (Desikan et al., 2006) was used to parcellate images into 34 regions per hemisphere. We extracted the three derived cortical measures: cortical thickness, surface area, and volume—calculated in FreeSurfer as a product of cortical thickness and surface area, though more accurate methods exist (Winkler et al., 2018) and are implemented in more recent versions of FreeSurfer (from version 6.0.0).

Following several reviewer suggestions regarding the role of MRI image parcellation, specifically the impact of region size on reliability, we also analysed data that had been processed using the Destrieux parcellation (Destrieux et al., 2010). These data were re-processed by Rutherford and colleagues to harmonise neuroimaging data from 82 sites (for complete details of data processing, see Rutherford et al., 2022). Freesurfer version 6.0 was used to extract cortical thickness from 74 regions per hemisphere, following the Destrieux parcellation.

#### 2.1.2 Participants

We included data from 7,269 participants (3,354 female, 3,915 male), for whom there were two available structural MRI scans. We removed 12 participants who belonged to a 22^nd^ site that was dropped from follow-up testing due to low numbers, as the low numbers would have hindered our multi-group analyses described below. Time from baseline to follow-up scan was on average 24.5 months (SD = 2.33) apart. Mean participants’ age at baseline was 9 years 11 months (range 9 years 1 month to 11 years 1 month), and at the 2 year follow-up was 11 years 11 months (range 10 years 5 months to 13 years 10 months).

The ABCD data re-processed from Rutherford et al. (2022) included 3670 participants (1728 female, 1942 male) for whom there were two available timepoints. Time from baseline to follow-up scan was on average 2 years 0 month (SD = 1.85). Mean age at baseline 9 years 11 months (range 9 years to 10 years 11 months), and at the follow-up was 11 years 10 months (range 10 years 7 months to 13 years 9 months).

### 2.2 ICED model

**Fig. 1.**
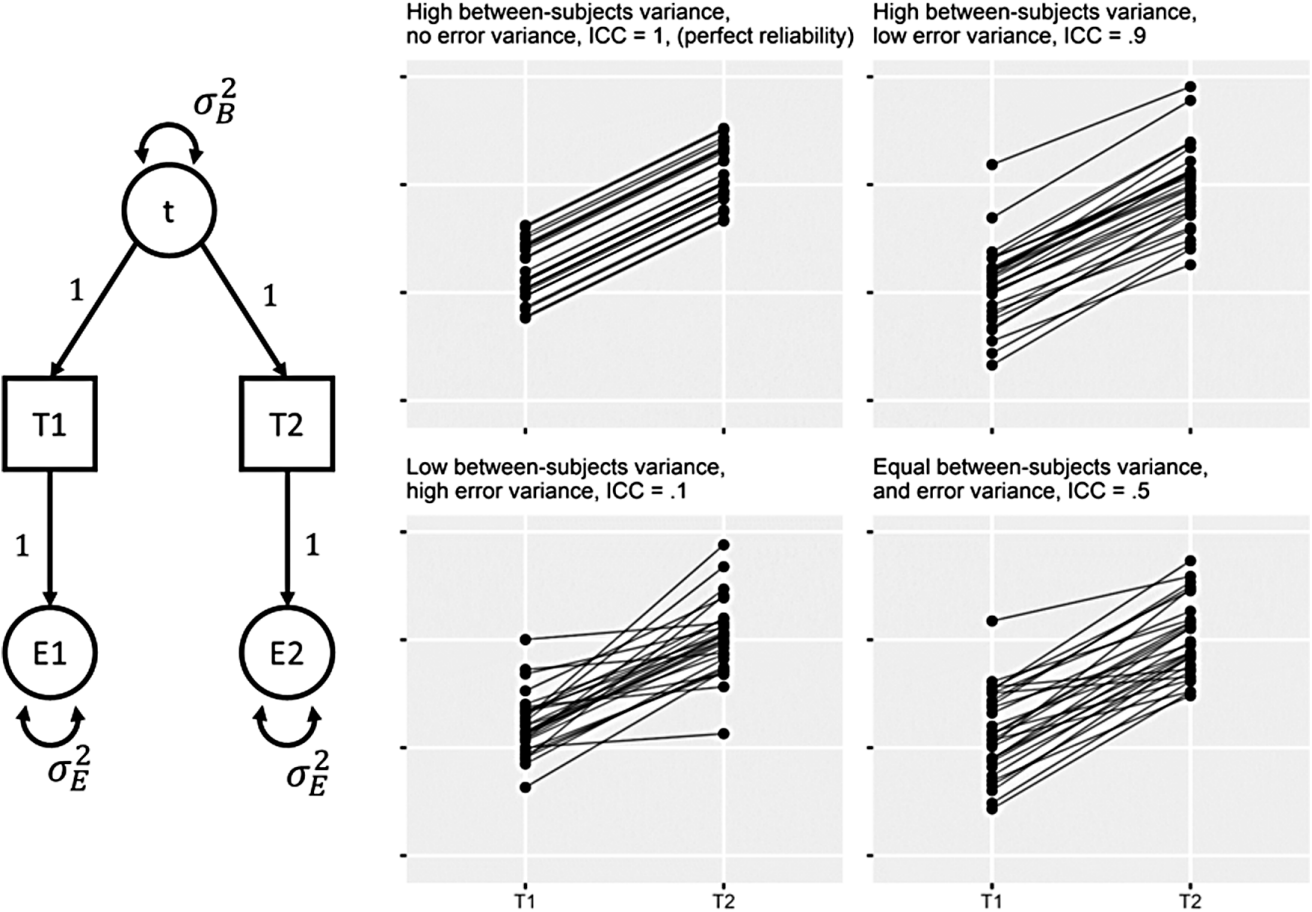
Left: Path diagram of the two timepoint ICED model used to estimate Between-subjects ($\sigma_{B}^{2}$) and error variance ($\sigma_{E}^{2}$) components. Right: Four plots visualising hypothetical differing levels of between-subjects and error variance, with equal total variance, to depict the relationship between test-retest reliability and rank-ordering individuals.

We used a two-timepoint ICED model implemented in the SEM framework (Brandmaier, Wenger, et al., 2018). Figure 1 (Left) presents a path diagram depicting the unique contribution of each source of variance. The two observed measurements are presented as rectangles, while the latent variables are presented as circles representing the sources of variance. Between-subjects variance ($\sigma_{B}^{2}$) captures variance attributable to individual differences between participants. Error variance ($\sigma_{E}^{2}$) captures the remaining variance that cannot be attributed to between-subjects differences, for example, within-subject fluctuations (hence sometimes being called *residual variance*). Single-headed arrows represent fixed regression loadings (set to 1), and double-headed arrows indicate the variance of the latent variables. The variance estimates for the two error latent variables (E1 and E2) were constrained to be equal.

Using this model, longitudinal stability is estimated as Intraclass Correlation Coefficients (ICCs), which are the most common measures that use test-retest reliability and longitudinal stability in neuroimaging research (Noble et al., 2021). ICC captures the reliability of an individual assessment. ICC is calculated using the between-subjects variance ($\sigma_{B}^{2}$) and error variance ($\sigma_{E}^{2}$) estimates as the proportion of between-subjects variance to total observed variance (Formula 1). Higher ICCs result from the between-subjects differences (i.e., individual differences) outweighing other sources of variance, in this case “error” (other sources of variance would be added to the denominator). Figure 1 (Right) presents four sets of simulated data to demonstrate the relationship between these two sources of variance and ICC estimates. One practical important take-home message from this figure is that test-retest reliability reflects how well we are able to rank-order participants and therefore how consistent this rank ordering is over time. In the first scenario (top left), if there were no measurement error, we would observe an ICC of 1, “perfect” reliability.^1^ Note that the rank ordering of participants remains the same over time. With near-perfect reliability (top right), there are some disruptions to the rank ordering, but overall we are very able to distinguish between individuals. With very low reliability (bottom left), there is very little consistency in the ordering of participants over time and we have little information with which we can distinguish between individuals. Between these two, when we have equal parts of between-subjects differences and error variances (bottom right), there is some consistency, but half of our signal (that we aim to use as a measure of individual differences) is unrelated to the construct we wish to measure. Note that across each simulation the total variance and the average difference over time is the same—we highlight the latter to reinforce that we are interested in between-subjects differences instead of differences in the mean over time (which may be the use of “stability” that some readers are more familiar with). To calculate ICC from our ICED model, we extract the between-subjects variance ($\sigma_{B}^{2}$) and error variance estimates ($\sigma_{E}^{2}$), and use this formula:

$$ICC=\frac{\sigma_{B}^{2}}{\sigma_{B}^{2}+\sigma_{E}^{2}}$$ (1)

Often we are interested in the reliability of the underlying construct, rather than individual indicators or observed measures. The estimate therefore takes into account the number of measurements—increasing the number of measures typically increases the reliability of the overall measure (e.g., Cronbach’s alpha or ICC). In the case of ICED models, the measurement structure is also incorporated into the model (e.g., capturing repeated measures nested within days; Brandmaier, Wenger, et al., 2018). To capture the construct-level reliability using this approach, we compute the *effective error* that would emerge as the residual error if we were to directly measure the construct. Effective error is derived from the power-equivalence theory (Brandmaier, von Oertzen, et al., 2018; Oertzen, 2010) and is a function of the combination of all sources of error (i.e., non between-subjects differences). Effective error can be calculated by generating a power equivalent model using the algorithm provided by von Oertzen (2010) or calculating a numerical estimate following the equations in Brandmaier, von Oertzen, et al. (2018) (Supplementary Material 3). This provides a flexible framework to calculate effective error for any complex study design. We can then calculate the construct-level reliability as ICC2 (Bliese, 2000), as follows:

$$ICC2=\frac{\sigma_{B}^{2}}{\sigma_{B}^{2}+\sigma_{EFF}^{2}}$$ (2)

where the between-subjects variance ($\sigma_{B}^{2})$ remains the same as in the ICC calculation. All other sources of variance are incorporated into the effective error term ($\sigma_{EFF}^{2}$). In our two timepoint models, consisting of between-subjects and error variance, $\sigma_{EFF}^{2}$ is calculated as $\frac{\sigma_{E}^{2}}{N}$ where N is the number of repeated measures (this follows from the multiple-indicator theorem (Oertzen, 2010)). From this, we can see that ICC2 will always show higher reliability relative to ICC, scaled by the number of independent measurements assuming measurements are independent and have identical variance. For example, our previous example of ICC = .5 corresponds to an ICC2 of .66 with two independent measurement occasions and an ICC2 of .75 with three independent measurement occasions. This reflects the improvement of reliability of an average score over repeated measurements by adding extra measurements. This is directly comparable to how one might improve the reliability of a questionnaire measure by increasing the number of items, for instance with reliability metrics like Cronbach’s alpha (Cronbach, 1951). Note that we follow prior ICED convention (Brandmaier et al., 2018) when referring to ICC and ICC2—these formulas correspond to a generalisable form of ICC3 and ICC3K following other conventions (see Koo & Li, 2016), allowing additional sources of variance. ICC3 defines “absolute” agreement across measures, that is, here that both time 1 and time 2 scans capture the same measurement. ICC3 then captures the reliability (or stability) of a single measure, while ICC3K captures the reliability (here longitudinal stability) of the mean of K number of repeated measures.

We used the R package *ICED* (Parsons et al., 2022; https://github.com/sdparsons/ICED) to run these analyses, which acts as a wrapper around the *lavaan* package (Rosseel, 2012)*.* Note that the Maximum Likelihood estimator assumes multivariate normality.

Additionally, ICED benefits from the powerful toolkit SEM offers that allow flexible modelling accommodating complex, nested study designs, including latent variables modelled by multiple indicators (e.g., left and right hemispheres as examined by Anand et al., 2022), (in)equality constraints, multigroup modelling, and model comparison techniques which allow for symmetric quantification of evidence for multiple competing models (Rodgers, 2010). We make extensive use of these in this study to capture distinct sources of variance and longitudinal stability.

### 2.3 Data analyses

To address our first question (what is the longitudinal stability of grey matter measures in the ABCD study, and do they differ across brain regions, structural metrics, and testing sites?), we ran a series of ICED models (Brandmaier, Wenger, et al., 2018; for other applied studies, see Anand et al., 2022; Wenger et al., 2021). We estimated between-subjects and error variances for three grey matter measures (cortical thickness, surface area, and volume) across regions of interest. We present test-retest ICCs to provide a “map” of test-retest stability across structural measures and brain regions. Following several reviewer suggestions, we also ran these analyses allowing the error variances at each timepoint to vary and compared the model fits. To address our second question (are these differences in longitudinal stability driven more by individual differences or measurement error?), we used a multigroup SEM and a series of model comparisons. We compared the relative influence of between-subjects variance and error variance across testing sites.

Given the challenges often associated with estimating such models, we implemented an approach that balances model optimisation and generalisability (proposed by Srivastava, 2018 and others). Specifically, we initially estimated the ICED modify the model on a randomly selected subset of all the data (495 participants), to make any necessary modifications to the model needed for estimation, prior to estimating the model on the full dataset (minus the initial exploratory subset). This ensures our final model estimation is more likely to converge and yield reliable estimation whilst being less likely to be overfit to the idiosyncrasies of a specific subset of the data. Based on this test-set, we multiplied surface area and grey matter volume by an arbitrary constant (.001) to ensure comparable variances across the three structural metrics.

## 3 Results

### 3.1 Stability estimates

**Fig. 2.**
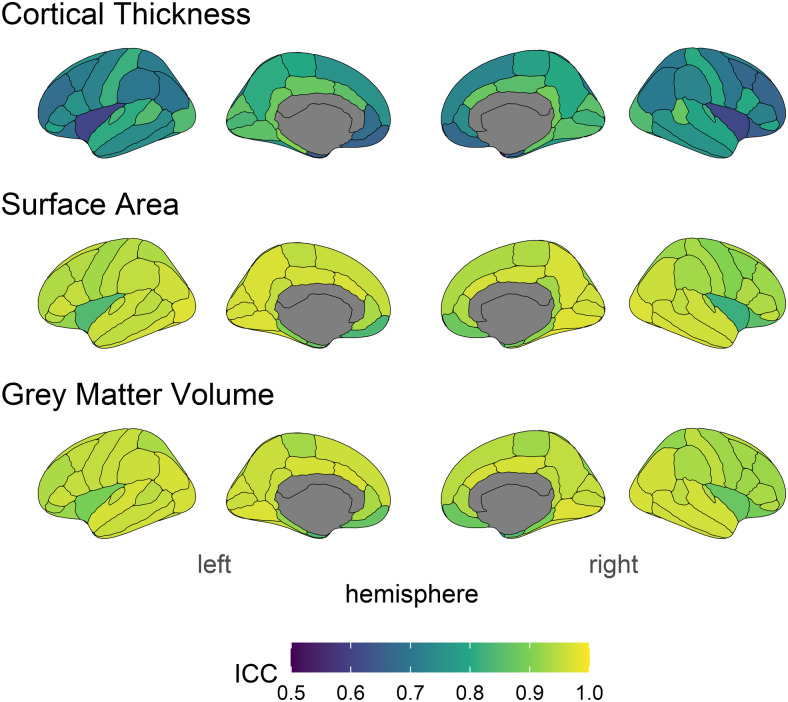
ICC estimates across structural measures and brain regions. Lighter colours indicate higher stability.

**Fig. 3.**
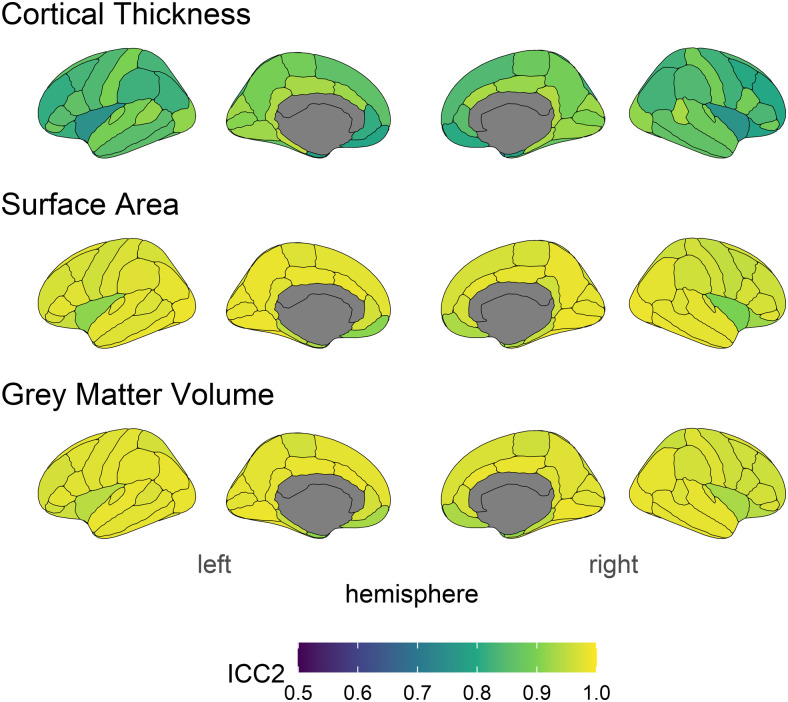
ICC2 estimates across structural measures and brain regions. Lighter colours indicate higher stability.

To estimate the longitudinal stability of grey matter measures, we fit our ICED model to each region across each structural measure. From each model, we extracted ICC and ICC2 estimates. Figures 2 and 3 visualise the ICC and ICC2 estimates, respectively, across measures and Desikan-Killiany Cortical Atlas (Desikan et al., 2006) regions of interest using the R package *ggseg* (Mowinckel & Vidal-Piñeiro, 2019).

ICC estimates the longitudinal stability of an individual indicator or measurement—essentially, how reliable do we expect a single measure to be? Mean ICCs for each measure were: cortical thickness .76 (range = .54 - .90; 95%CI widths ranged from .012 to .048 around ICC), surface area .93, (range = .82 - .97; 95%CI widths ranged from .005 to .068 around ICC), and volume (mean = .93, range = .76 - .97; 95%CI widths ranged from .005 to .029 around ICC). Estimates for each brain region and measure can be found in the Supplementary Materials. Comparing measures, cortical thickness showed an overall poorer pattern of longitudinal stability. While all estimates for surface area and volume above commonly used cut offs for “good”^2^ longitudinal stability (>.75), for cortical thickness 46% of regions had stability lower than .75^2^. This relatively low longitudinal stability of this measure means that true patterns or associations will likely be attenuated and/or rendered non-significant purely because of lower stability estimates. We discuss these practical implications in more detail below.

The ICC2 provides an estimate of longitudinal stability at the level of the construct. ICC2 estimates (Fig. 3) show the same pattern of ICCs across brain regions, albeit higher values. The mean ICC2s for each measure were: Cortical Thickness = .86 (range = .70 - .95), surface area = .96 (range = .90 - .99), and volume = .96 (range = .86 - .99).

As several reviewers suggested, it is plausible that the variances of cortical measures may differ between timepoints, perhaps due to developmental factors, acclimation. Therefore, as an additional exploratory analysis we performed these analyses again allowing the error variances at each timepoint to vary. We compared the comparative fit index (CFI; (Bentler, 1990) for the constrained (i.e., constraining the error variances to be equal across time points) and unconstrained models (i.e., allowing the error variances to vary across time points). Briefly, the CFI is an incremental fit indicating better model fit and a CFI greater than .96 (Hu & Bentler, 1999) is typically used to indicate good fit (CFIs cannot exceed 1). A difference in CFI between two models greater than .02 is typically used as an indication one model has meaningfully better fit (Meade et al., 2008). Note that the unconstrained models are saturated with zero degrees of freedom and thus CFI always equals 1. Meaningfully poorer model fit in the constrained models indicates that strict measurement invariance does not hold and an alternative approach to ICC longitudinal stability may be warranted. Only two brain regions (the Supramarginal gyrus in both hemispheres), and only for cortical thickness, had a difference in CFI greater than .02. Further research may benefit from direct examination of these regions. However, for the remainder of our analyses, we continue to constrain the error variances at both time points to be equal.

### 3.2 Examining sources of longitudinal (in)stability

To probe potential variability in stability across additional factors, we re-ran the ICED model across each of the 21 sites, again separately for each brain region. For brevity, and because Cortical Thickness showed the largest heterogeneity in ICC across brain regions, we present results from Cortical Thickness only (analysis output and figures for surface area and grey matter volume can be found in the Supplementary Materials). We then decomposed these longitudinal stability estimates into the between-subjects and error variance components. This allowed us to quantify the relative contributions of both variance components across brain regions and testing sites.

#### 3.2.1 Region differences

**Fig. 4.**
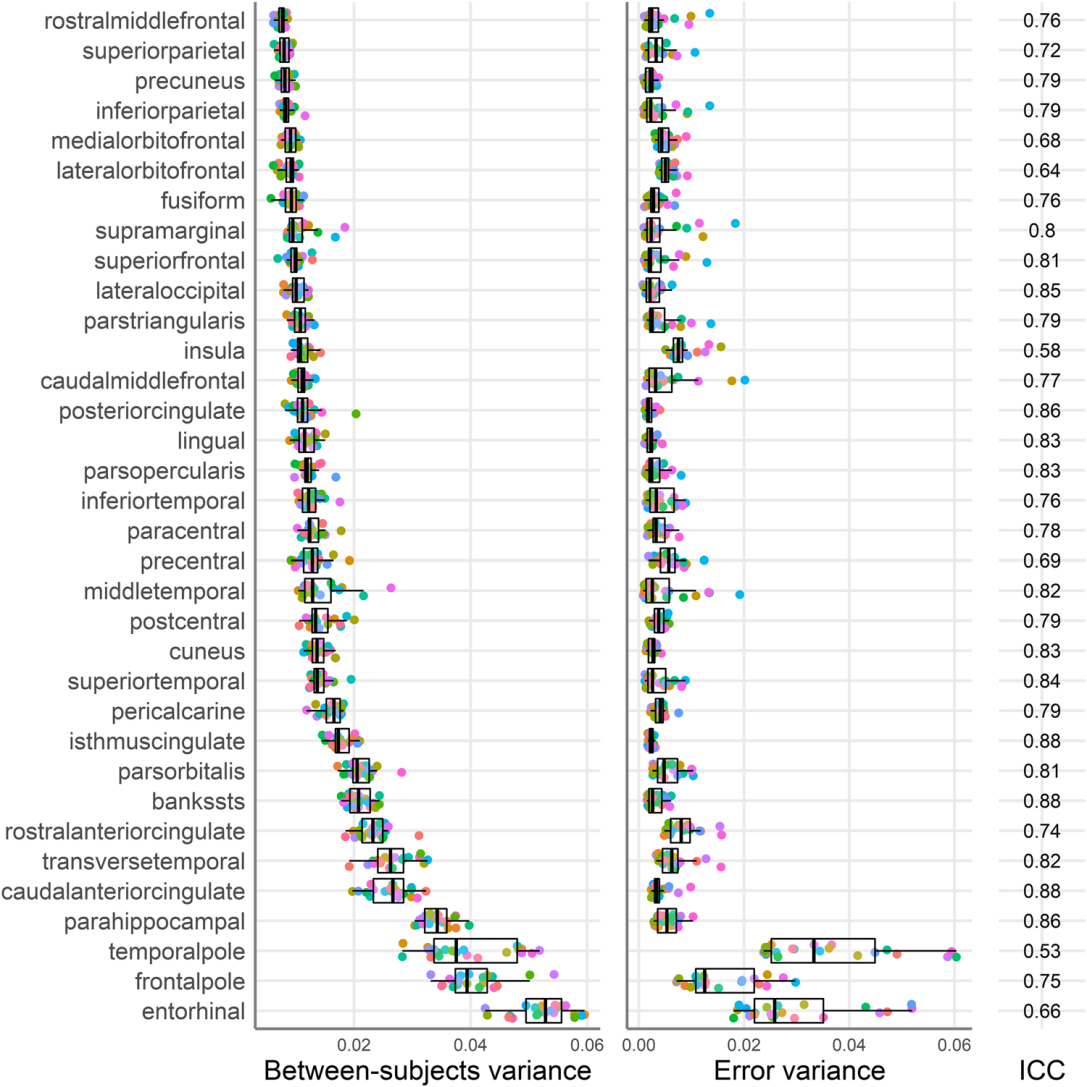
Between-subjects (left panel), error variance estimates (middle panel), and median ICC (right panel) for each region of interest (y-axis). Regions are ordered by the median between-subjects variance. For clarity we present only the right hemisphere regions. Each point represents a different testing site, and the colour mapping is the same as in Figure 5. The boxplots present the median and the 25^th^ and 75^th^ percentiles, the whiskers extend at a maximum to 1.5 times the interquartile range from the box.

To explore the sources of differences in stability estimates across brain regions, we compared the relative size of between-subjects and error variances across each brain region. Figure 4 plots the between-subject (left panel) and error variance estimates (right panel) for each region of interest, with each point representing a different testing site. As expected from a visual inspection of Figure 4, on average, the variance of the between-subjects variance estimates was 2.8 times larger than the error variance estimates. This suggests that differences in stability estimates across regions are likely driven more by differences in the between-subjects variance than site differences in measurement error.

#### 3.2.2 Site differences

**Fig. 5.**
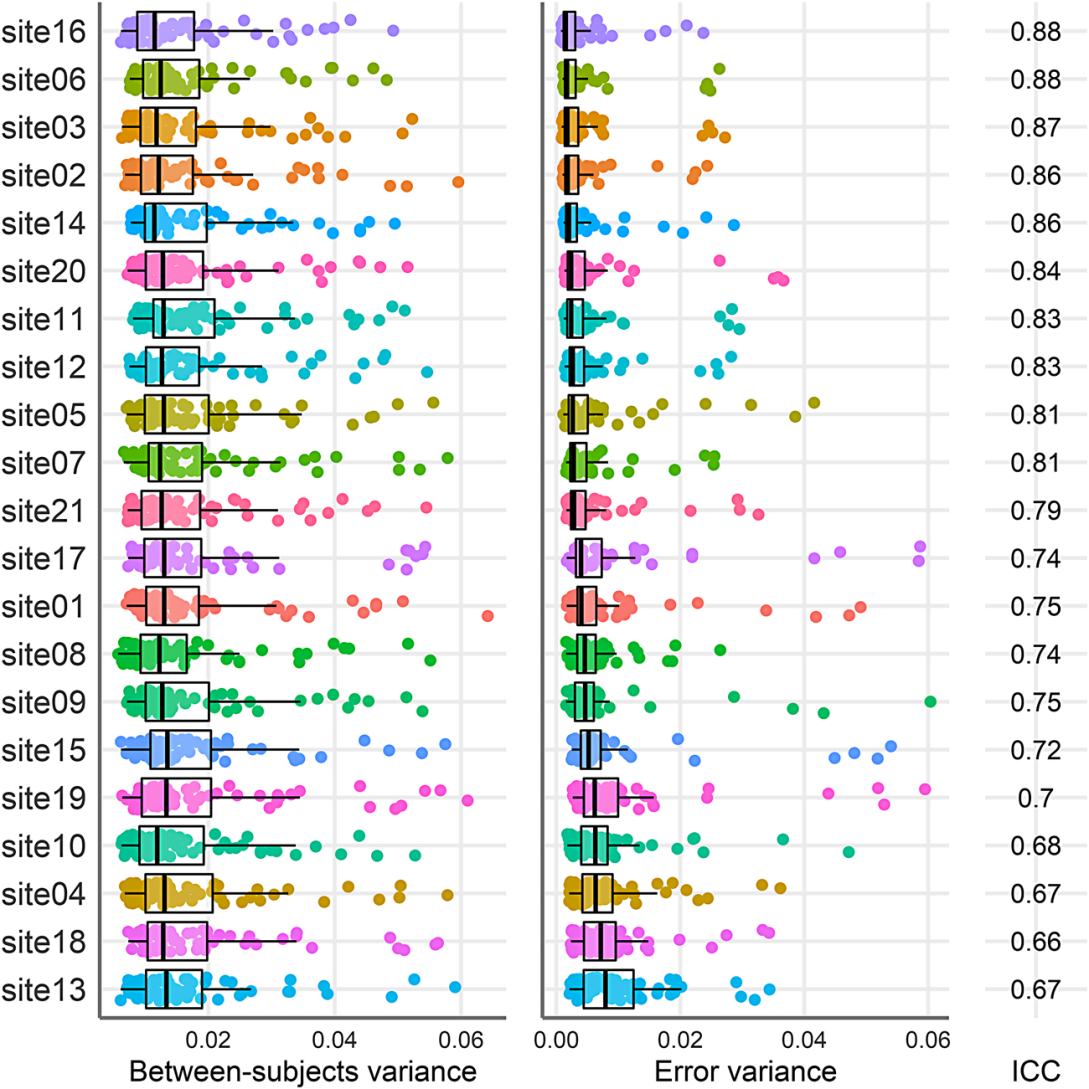
Between-subjects (left panel), error variance (middle panel) estimates, separately per testing site. Sites are ordered by the median error variance. Each point represents a different brain region, and the site colour maps to Figure 4. Cortical thickness only. The boxplots present the median and the 25^th^ and 75^th^ percentiles, the whiskers extend at a maximum to 1.5 times the interquartile range from the box.

Figure 5 plots the latent between-subject and error variances across brain regions separately for each site. In contrast to Figure 4, the distributions of between-subject variance estimates are largely overlapping across sites. In contrast, the distributions of error variance differ markedly across sites in both the median estimate and the interquartile ranges. To help quantify the difference in contributions from between-subject and error variance, we extracted the median variance estimates for each region and calculated the variance of these estimates to compare the spread of between-subject variance and error variance. Across sites, there was 11.5 times more variance in the median error variance estimate than the median between-subject variance estimate. This suggests that differences in stability across testing sites are driven mainly by differences in error across sites, rather than genuine differences between people in each location. We later discuss potential causes of these differences in error.

#### 3.2.3 Rank order stability of ICC estimates

To assist interpretation, we also calculated rank order stability of ICC, between-subjects variance, and error variance estimates. We did this separately for region differences and site differences, allowing us to capture the extent to which the same region, or the same site, is (un)reliable. Table 1 reports ICC (2,1) and ICC (3,1) estimates (Koo & Li, 2016). ICC(3,1) indexes *consistency agreement* and can be conceptualised as the degree to which scores can be equated to each other, with some systemic error. ICC(2,1) is a more conservative index of *absolute agreement* across measures that additionally penalises for any systemic error. To illustrate, consider two repeated measures for which participants score the exact same number (Time1 = Time2). Here, we have perfect longitudinal stability, both ICC(2,1) and ICC(3,1) equal 1. Now, consider instead that due to some practice effects all participants score 2 points higher in the second measure (Time1 = Time2 - 2). Here, ICC(3,1) = 1, indicating perfect longitudinal stability, while our ICC(2,1) will be lower as a result. These estimates give an indication of whether the stability estimates for brain regions are consistent across testing sites, and whether the same testing sites are consistently more or less reliable across brain regions.

**Table 1.** ICC2,1 and ICC3,1 estimates separately comparing the rank-order stability of site and brain region estimates of ICC, between-subjects variance, and error variances.

	By region: How stable are regional estimates across sites?		By site: How stable are site estimates across regions?	
	ICC2	ICC3	ICC2	ICC3
ICC	0.45	0.64	0.30	0.54
Between-subjects variance	0.94	0.95	<0.01	0.07
Error variance	0.76	0.82	0.07	0.30

The rank-order stability of brain region estimates (ICC, between-subjects variance, and error variance) suggests that across testing sites the same brain regions tend to have higher, or lower, longitudinal stability. Supporting our previous analyses, it is particularly clear that different brain regions typically have differing levels of between-subjects variance. In contrast, the rank-order stability of site estimates is considerably lower (particularly for the variance estimates), suggesting that we cannot discern that particular testing sites show higher or lower longitudinal stability across brain regions.

#### 3.2.4 Multigroup models for site differences

**Fig. 6.**
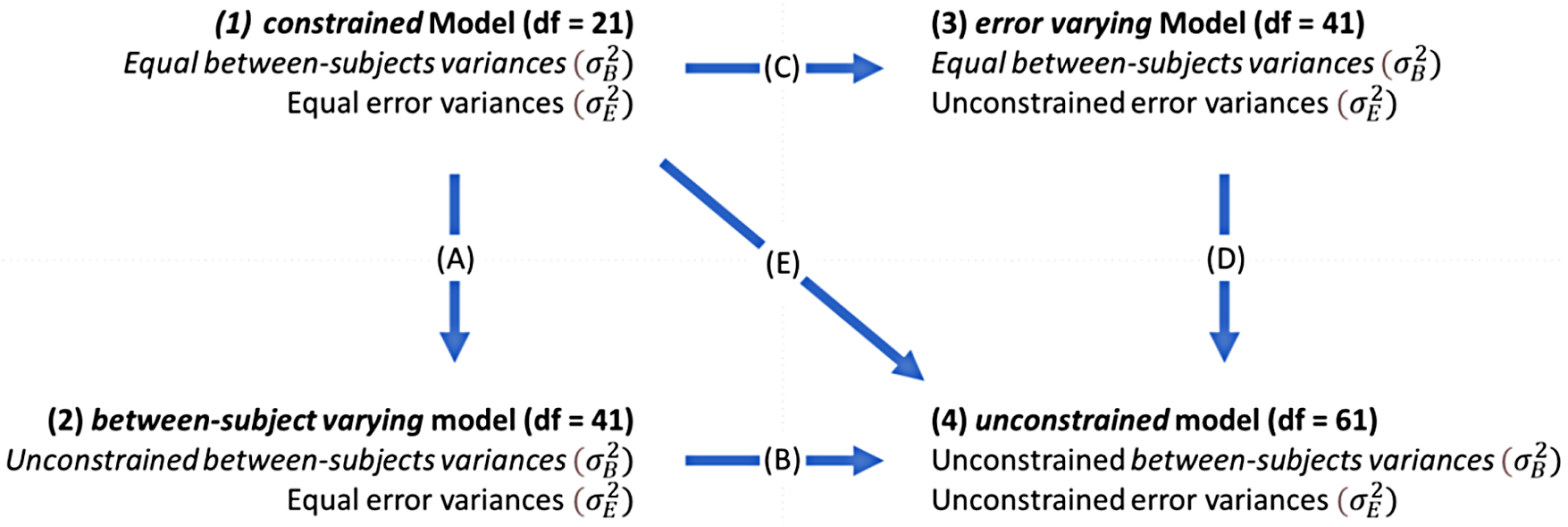
Representation of multigroup ICED models (numbers 1-4), and model comparisons (arrows A-E). The model descriptions refer to whether the between-subjects variance ($\sigma_{B}^{2}$) and error variance ($\sigma_{E}^{2}$) parameters were allowed to vary across sites (unconstrained) or were set to be equal across sites (equal). The arrows represent the model comparisons (ΔCFI) in the direction towards the less constrained model.

To more formally assess potential cross-site variation in stability across measures and brain regions, we performed a series of four multigroup ICED models, such that each site is represented by a different group. Specifically, the four models were (1) a *constrained* model, in which all groups were constrained to have equal between-subjects and error variances; (2) a *between-subjects varying* model in which the between-subjects variance parameter was free to vary across groups (while we set an equality constraint on the error variance parameter across groups); (3) an *error varying* model in which the error variance parameter was free to vary between groups (while we set an equality constraint on the between-subjects variance parameter across groups); and (4) an *unconstrained* model in which both variance components were allowed to vary between groups. Including comparisons with the between-subjects and error-varying models allows us to make some inferences about the sources of differences in stability across sites—that is, whether stability differences across sites are due to different levels of between-subjects differences or measurement error. To compare model fit, we extracted the Comparative Fit Index (CFI; Bentler, 1990) for each model and computed the difference in CFI (ΔCFI) for five model comparisons: (A) constrained—between-subjects varying, (B) between-subjects varying—unconstrained, (C) constrained—error varying, (D) error varying—unconstrained, and (E) constrained—unconstrained. Figure 6 presents the models and model comparisons visually. Greater ΔCFI values indicate larger improvements in model fit for the less-constrained model. ΔCFI values greater than .02 (Meade et al., 2008) have been proposed as thresholds to determine differences in fit.^3^

**Fig. 7.**
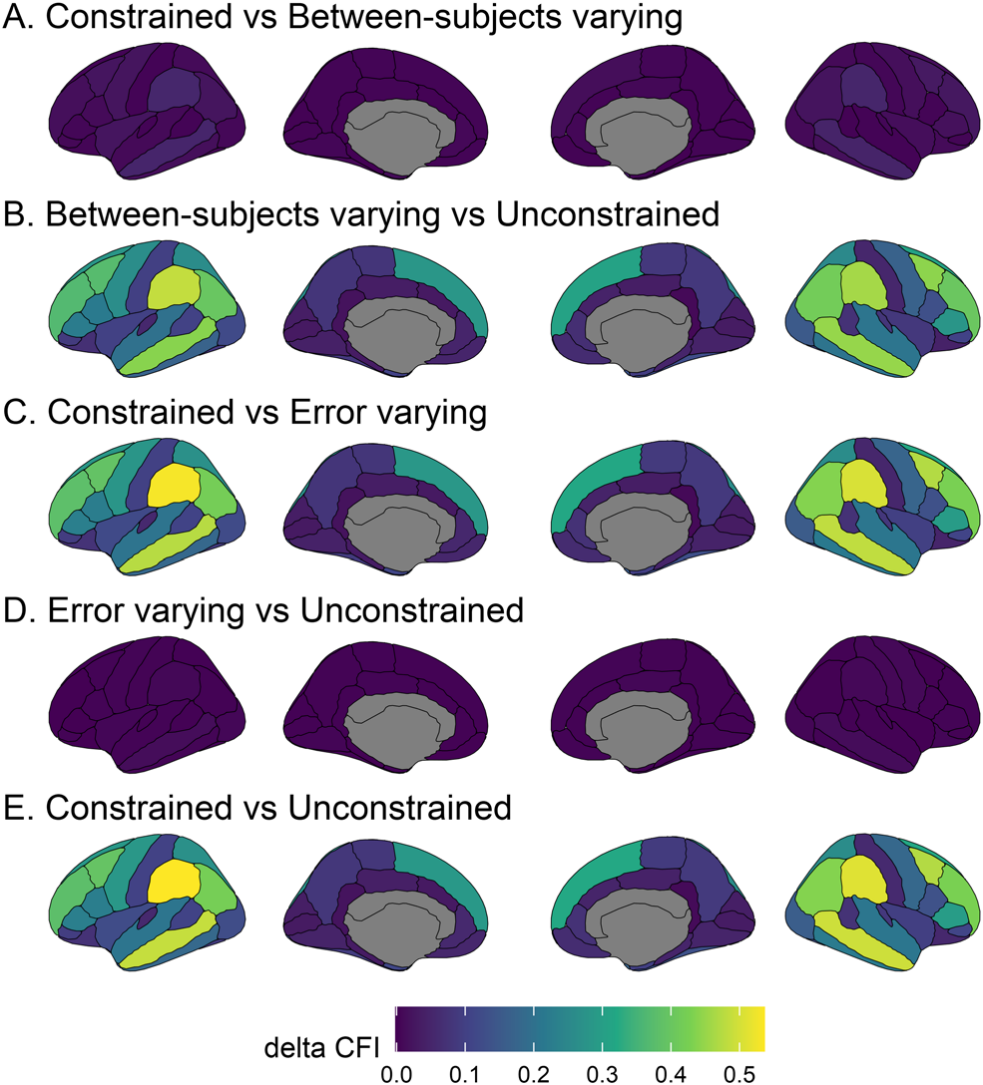
ΔCFI for each model comparison (A-E) across regions. Higher values (lighter and more yellow coloured) indicate improved model fit with more free parameters. In comparisons A and D, between-subjects variance is allowed to vary compared to the preceding model. In comparisons B and C, error variance is allowed to vary compared to the preceding model. In panel E, both between-subjects and error variances are allowed to vary compared to the fully constrained model.

Figure 7 presents ΔCFI values for each model comparison across each brain region. Higher values indicate that the more complex model (with more free parameters) better fit the data even when penalizing for the additional complexity. Allowing the error variance to vary across sites (comparisons B, C, and E) meaningfully improved model fit in almost all cases (ΔCFI greater than .02 in over 97% brain regions). This suggests that testing sites are characterised by differing levels of measurement error. In contrast, allowing between-subjects variance to vary across sites (comparisons A and D) typically led to negligible or negative (1.5% of brain regions in comparison A and 13.2% of brain regions in comparison D) improvements in model fit, thus favouring the more parsimonious model, suggesting between-subjects variance did not differ systematically between sites. Allowing between-subjects variance to vary across sites improved the fit (ΔCFI greater than .02) in 19% of brain regions compared to the fully constrained model (comparison A) and in 0 regions compared to the error varying model (comparison D). This suggests that the between-subjects variance components are highly similar across testing sites and allowing between-subjects variances to vary across sites does not improve model fit over allowing error variances to vary across sites.

#### 3.2.5 Follow-up multigroup analyses by scanner manufacturer

We expanded these analyses to explore the influence of MRI scanner on between-subjects variance and error variance. We ran the series of multigroup models and model comparisons described above, treating MRI scanner manufacturer (Siemens, 13 sites; Philips Medical Systems, 3 sites; and GE Medical Systems, 5 sites) as the grouping variable. From these analyses, we generated Figures 4, 5, and 7 for each metric (cortical thickness, surface area, and volume) and provide these in the Supplementary Material. For Cortical thickness; scanners from Siemens, Philips Medical Systems, and GE Medical Systems had average ICCs across brain regions of .83, .72, and .69, respectively. The multigroup model comparisons also showed a near identical pattern of results (Figure7_CT_scanners in the Supplementary Material) as those presented above treating testing site as the grouping variable (Fig. 7).

We then ran three series of multigroup models by site (as described in the previous section), separately for the sites with each scanner manufacturer (Supplementary Figures: Figure7_CT_Siemens, Figure7_CT_Philips, and Figure7_CT_GE). For each scanner manufacturer, allowing between-subjects variance to vary between sites did not generally improve model fit—matching the general pattern of results. However, the patterns of results for allowing error variance to vary between sites differed markedly across brain regions within each scanner manufacturer. Together, these patterns of results suggest that there are both scanner and site-level influences on the amount of error variance in our measures of grey matter, and that these influences differ across brain regions.

### 3.3 Practical implications

Above, we quantified the longitudinal stability of three grey matter measures. We can use these estimates to answer pragmatic questions about study design choices, including: how many repeated brain scans do we need to achieve high longitudinal stability? And, what influence are differences in longitudinal stability across brain regions likely to have on the attenuation of our results?

#### 3.3.1 How many repeated measures do we need to achieve high longitudinal stability?

We answered this question assuming that the stability estimates are proxies for reliability estimates. To put these estimates into context, we performed a brief decision-study (Shavelson & Webb, 1991; Vispoel et al., 2018; Webb et al., 2006), using the Cortical Thickness estimates. We estimated the number of repeated measures needed to achieve an ICC2 longitudinal stability of greater than .9—“excellent” longitudinal stability, following Koo and Li’s standards (2016). We can reformulate the ICC2 formula for this purpose.

$$ICC2=\frac{\sigma_{B}^{2}}{\sigma_{B}^{2}+\frac{\sigma_{E}^{2}}{N}}$$ (3)

$$N>\frac{ICC2\cdot \sigma_{E}^{2}}{\left(1-ICC2\right)\cdot \sigma_{B}^{2}}$$ (4)

Then, for ICC2 > .9

$$N>\frac{9\cdot \sigma_{E}^{2}}{\sigma_{B}^{2}}$$ (5)

**Fig. 8.**
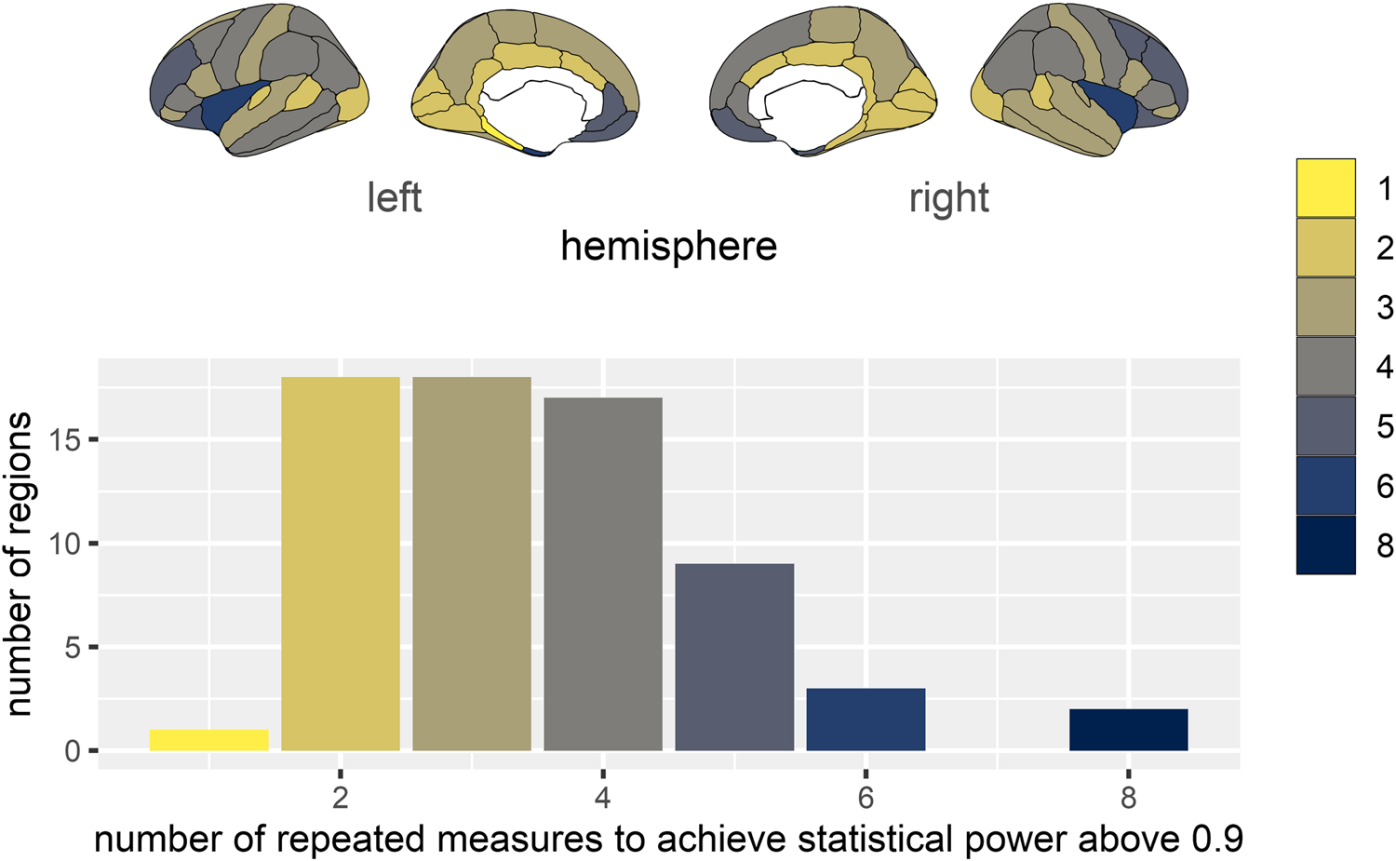
Number of timepoints required to achieve an ICC2 longitudinal stability estimate of .9 or greater for Cortical Thickness brain regions (assuming no individual differences in change in cortical thickness over 2 years).

As visualised in Figure 8, our estimates suggest that most (48 of 68, or 70.5%) regions would require three or more timepoints to achieve an ICC2 longitudinal stability greater than .9. Further, 45.6% regions would require four or more timepoints. Performing poorest were the left and right temporal pole regions—both would require eight repeated scans to achieve high longitudinal stability. Given there are relatively few longitudinal brain imaging studies (Kievit & Simpson-Kent, 2020), and most of these contain only two timepoints, these results suggest that we will be unlikely to achieve sufficient longitudinal stability in some brain regions. Substantively, our findings therefore suggest that the absence of findings in these regions in similarly designed studies may therefore reflect low power (caused by suboptimal longitudinal stability) rather than a true absence of effects or differences between individuals or groups.

Also note, with repeated measures within session, we are likely to improve the longitudinal stability of our measurements. Further, it is possible to use data with repeated measures within session to estimate the contribution of these additional components of variation. Using these variance components, we can perform a similar decision study as above to investigate the benefits (and any related cost-benefit trade-offs) of including additional within-session measurements (Anand et al., 2022; Brandmaier, Wenger, et al., 2018; Noble et al., 2017).

#### 3.3.2 How attenuated are our estimates likely to be?

A related practical implication is that our standardised effect sizes will be more attenuated for regions with lower longitudinal stability. To demonstrate this, we extracted estimates from regions with the highest (parahippocampal gyrus, left hemisphere ICC = .9) and lowest (temporal pole, right hemisphere ICC = .54) Cortical Thickness longitudinal stability estimates. For example, assuming a “true” correlation between a hypothetical measure and each brain region is .3, and the hypothetical measure has a longitudinal stability of .9.

$$r_{observed}=r_{truecorrelation}\sqrt{r_{measure}\times r_{region}}$$ (6)

$$r_{observed\left(\text{parahippocampal gyrus}\right)}=.3\sqrt{.9\times .9}=.27$$ (7)

$$r_{observed\left(\text{temporal pole}\right)}=.3\sqrt{.9\times .54}=.21$$ (8)

Using Spearman’s attenuation correction formula (Equation 6; Spearman, 1904), we expect the parahippocampal gyrus correlation to be attenuated to .27 (equation 7) and the temporal pole to be attenuated to .21 (equation 8). We can use these attenuated effect size estimates to compare expected statistical power for a straightforward correlation analysis. Given the attenuation, we would require almost 70% more participants (105 vs. 175) to detect the more severely attenuated correlation with 80% statistical power with a 5% alpha.

### 3.4 The relationship between size of brain region and longitudinal stability

Above, we have focused on the Desikan-Killiany-Tourville atlas parcellation (34 regions per hemisphere) as it is very widely used within and beyond ABCD, and thus will allow researchers to directly compare patterns of empirical findings with patterns of reliability. However, it is also highly plausible that different atlases will yield different measures of reliability even in the same sample for reasons of region size (averaging out noise to differing degrees) and anatomical fidelity^4^*. To that end, we have now added an extensive analysis based on combining regions into lobes (5 lobes per hemisphere) and a custom Destrieux parcellation (74 regions per hemisphere).

#### 3.4.1 Lobes analysis

**Fig. 9.**
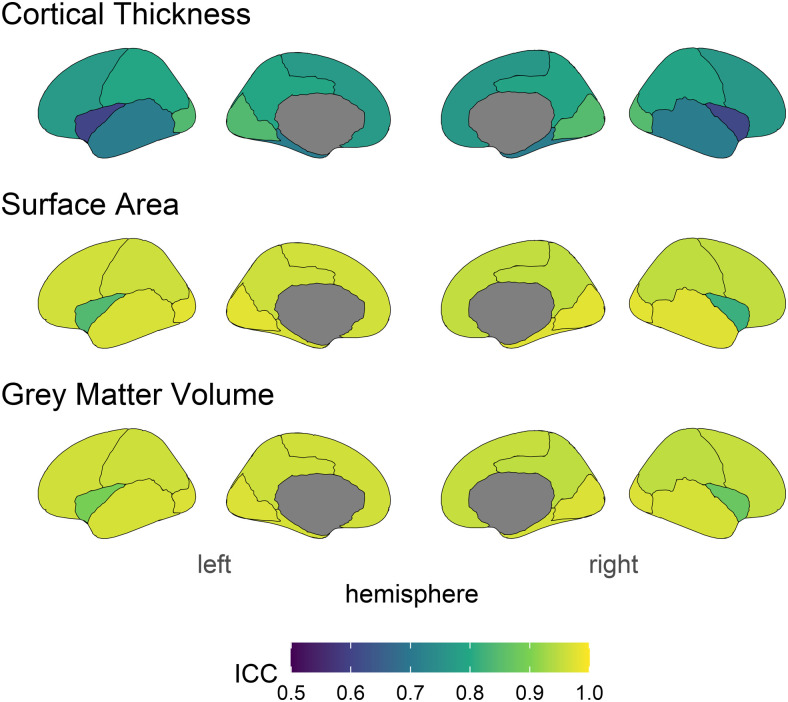
ICC estimates across structural measures and lobes. Lighter colours indicate higher longitudinal stability.

We reran the ICED models to estimate ICC longitudinal stability on lobes. We combined lobes following freesurfer guidelines (Klein & Tourville, 2012, Appendix 1), calculating the mean cortical thickness, and sum surface area and volume for each lobe. Figure 9 visualises the ICC for each lobe across each measure. Mean ICCs for each measure were: cortical thickness .74 (range = .6 - .84), surface area .93 (range = .82 - .98), and volume (mean = .95, range = .87 - .97). To compare the ICCs based on lobe and the ICCs based on individual regions, we calculated the mean ICC across brain regions for each lobe. The lobe ICCs were marginally larger: difference in cortical thickness was .00 (range -.05 to .04), difference in surface area was .02 (range .00 to .04), and difference in volume was .02 (range .00 to .05). Finally, following the exploratory analysis above, we compared the model fit for models with constrained and unconstrained error variances. The CFI was not meaningfully poorer in the unconstrained model for any lobe across the brain measures, indicating that we can model variances equally at each timepoint.

#### 3.4.2 Destrieux parcellation analysis

**Fig. 10.**
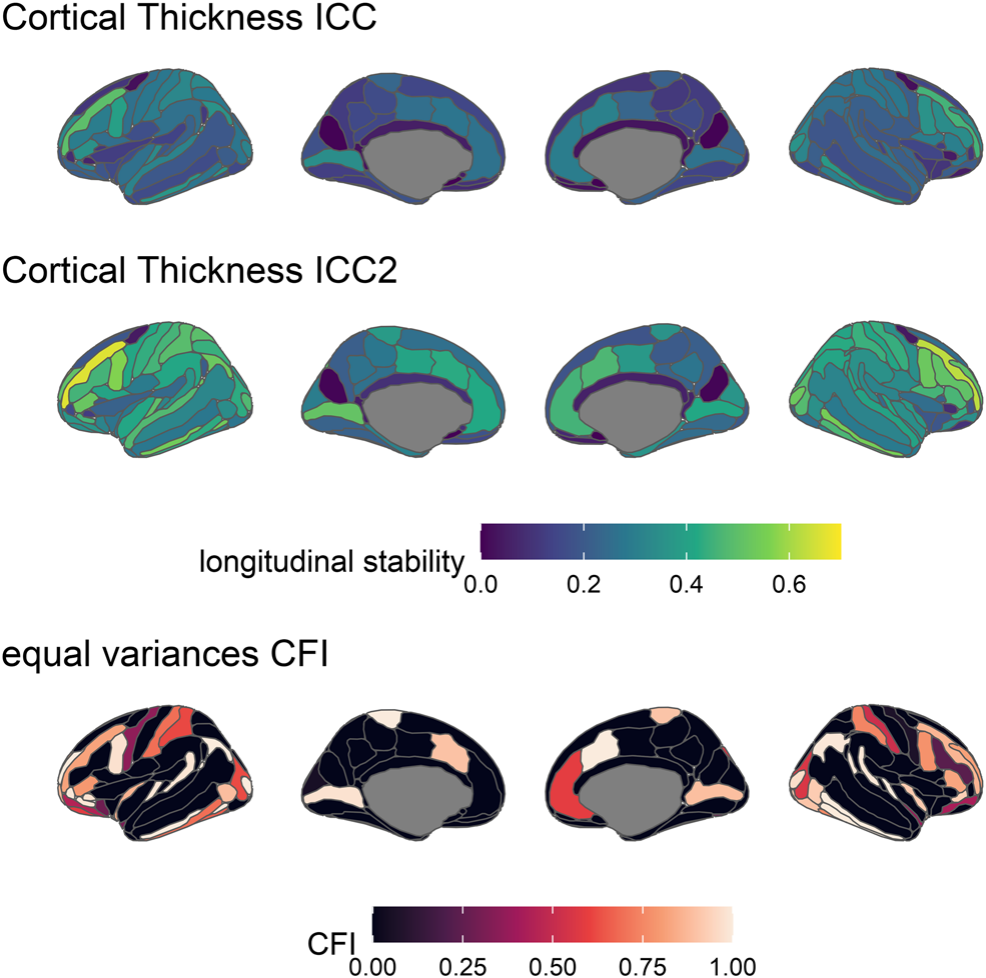
ICC (top), ICC2 (middle), and CFI estimates for the constrained (error variances equal between timepoints) ICED model (bottom) for cortical thickness across brain regions (Destrieux parcellation). For ICC estimates (top and middle), lighter colours indicate higher stability. Note that for clarity we shifted the scale compared earlier in ICC (Figs. 2, 3, and 9). For CFI estimates (bottom), lighter regions indicate higher CFIs and better model fit (above .95 is desirable to accept the model), black regions indicate a CFI of zero indicating terrible model fit and that we cannot trust the model to yield reliable estimates.

Next, we ran our ICED models on data processed using the Destrieux parcellation (Rutherford et al., 2022). Figure 10 visualises the ICC, ICC2, and model fit (CFI) of the ICED models. The mean ICC for cortical thickness across Destrieux brain regions was .20 (range .00 to .50), and the mean ICC2 was .32 (range .00 to .66). We again compared the model fit for models with constrained and unconstrained error variances. In contrast to our analyses using the Desikan-Killiany-Tourville atlas (Desikan et al., 2006), only 13% of regions (19 regions), the CFI difference did not favour the constrained model indicating that for most regions we cannot model error variances to be equal across timepoints. This indicates a violation of strict measurement invariance over time for these regions, and that alternative longitudinal analytic approaches may be needed (though for an in-depth discussion of the role of measurement invariance see Robitzsch & Lüdtke, 2023). We also found poor model fit in the constrained ICED models across most regions: in 57% of brain regions, the CFI was 0 and in 87% of cases the CFI was lower than .95 (usually a lower threshold for acceptable fit).

**Fig. 11.**
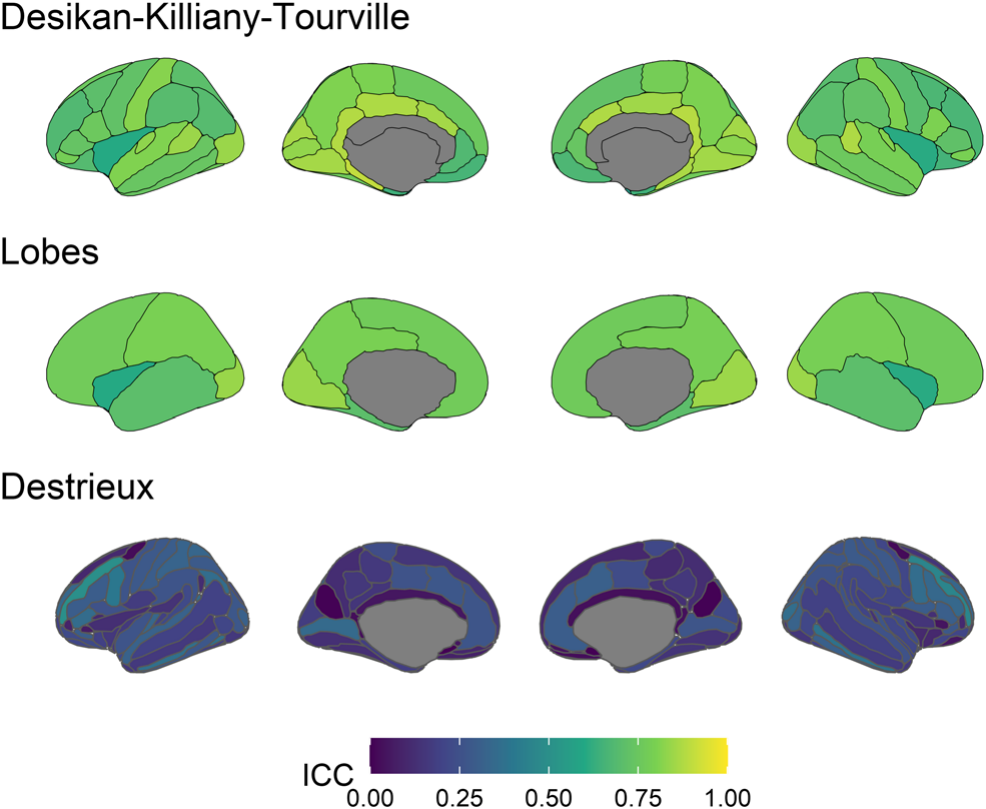
ICC estimates for cortical thickness for the Desikan-Killiany-Tourville parcellation (top), the combined lobes from the Desikan-Killiany-Tourville parcellation (middle), and the Destrieux parcellation (bottom). Lighter colours indicate higher longitudinal stability.

To aid comparisons of results, Figure 11 presents the ICC estimates for cortical thickness only for the Desikan-Killiany-Tourville parcellation, the combined lobes, and the Destrieux parcellation using the identical colour mapping.

## 4 Discussion

In this study, we used a series of ICED models (Brandmaier, Wenger, et al., 2018) to generate brain maps of (2-year) longitudinal stability to provide a nuanced overview of the stability of grey matter across imaging measures and brain regions in the ABCD study imaging data (Casey et al., 2018). Our first analyses demonstrated heterogeneity in longitudinal stability estimates of longitudinal stability across brain regions. Further, of the grey matter structural measures (thickness, surface area, and volume), “one of these is not like the other.”^5^ Specifically, cortical thickness showed a lower average longitudinal stability, and a wider range of longitudinal stability estimates, across brain regions. In contrast, surface area and grey matter volume showed near identical patterns of high longitudinal stability.

The low longitudinal stability we see in some regions may simply be because those regions are harder to image. For example, the inferior temporal cortex and frontal poles are close to regions susceptible to various artifacts, including the temporal bones, sinuses, and potential dental artifacts. Indeed, these same regions have previously been found to have low test-retest reliability (Knussmann et al., 2022).

The lower stability of cortical thickness may either reflect true lower reliability, but also the greater individual differences in true cortical thickness change known to occur in this developmental period. Repeated scans closely spaced (i.e., hours or days apart) in a developmental cohort would allow future researchers to disentangle these explanations. During early adolescence, surface area and volume are relatively stable while cortical thickness is rapidly changing (Bethlehem et al., 2022; Mills et al., 2016; Rutherford et al., 2022). The fact that cortical thinning is occurring does not itself account for differences in longitudinal stability. The ICED estimated ICC does not penalise for change over time, assuming that all participants are changing at the same rate. However, individual differences in the *rate of change* will lead to reduced longitudinal stability as these individual differences will be included in the error term if not explicitly modelled. Individual differences in the rate of cortical thinning are well documented (Bethlehem et al., 2022; Rutherford et al., 2022), including quantifications of cortical maturation (Fuhrmann et al., 2022), and are associated with pubertal timing (Vijayakumar et al., 2021), itself an important source of intra-individual differences across adolescents. Together, there are exciting possibilities to investigate and quantify how rapid changes in brain structure—including during sensitive developmental periods including adolescence (Fuhrmann et al., 2015) and in later life—influence longitudinal stability. Below, we discuss opportunities to expand ICED into the latent growth curve model to incorporate these individual differences in the rate of change.

We extended our analyses to examine the relative contributions of between-subjects variance and error variance on differences in patterns of stability across brain region and ABCD’s 21 testing sites. Stability estimates were heterogeneous across regions, and this appeared to be driven by differences in between-subjects variance, suggesting these differences are due more to actual between-subjects differences. This observation is encouraging insofar as we can be more certain that observing individual differences across brain regions is likely a result of those individual differences, instead of differences in the amount of error captured in each region (perhaps with exceptions of the temporal pole, frontal pole, and entorhinal cortex).

In contrast, we found that differences in longitudinal stability across testing sites were largely driven by differences in error variance. It is not yet clear why some sites contribute more error than others. The ABCD consortium has gone to great lengths to ensure consistency in scanning parameters, data processing, quality control, and data harmonisation (Casey et al., 2018; Hagler et al., 2019). In our follow-up analyses, we found the average contribution of error variance differed between scanner manufacturers, and an overall similar pattern of results in the multigroup analyses. We also saw differing patterns of site-related differences in error variances when analysing sites with each scanner type separately. Given the study design, with sites using a single scanner type, we are unable to fully disentangle the contributions of site-related and scanner-related influences on error. It may be possible to test this with a multilevel ICED model, ideally on a dataset with cross-nesting of site and scanner, and we welcome extensions of our work in this area. In addition to site-related differences (e.g., MRI scanner and image acquisition), site-related sampling differences, including demographics like age, related to each site may also be impactful. It is also plausible that sites were differentially affected by recruitment, retesting, and COVID-19 related delays. We expect that time between scans moderates the longitudinal stability and stability of the measures (as discussed in the introduction). At the site level, different patterns of time lags between scans may capture differing levels of individual differences in change over time—which in these models would lead to higher estimated error.

We included two follow-up exploratory analyses. First, we investigated the influence of cortical atlas parcellation on longitudinal stability, combining data across Desikan-Killiany-Tourville regions into lobes and analysing data processed with the Destrieux parcellation. We found the Destrieux parcellation yielded far lower longitudinal stability ICC estimates (mean = .20, range = .00 to .50) than for the Desikan-Killiany-Tourville parcellation (mean = .76, range = .54 to .90; also see Fig. 11). Although we cannot definitively attribute these differences to the atlases in question, it seems likely that authors should be mindful of the relative strengths and weaknesses of each atlas. Greater anatomical fidelity may be associated with lower reliability for a range of reasons—the optimal choice will vary depending on the goal of the study. Second, we investigated whether error variances across timepoints can be constrained to equality. For the Desikan-Killiany-Tourville parcellation, the only regions for which allowing error variances to differ improved model fit were for the cortical thickness of the supramarginal gyrus (both hemispheres). For the Destrieux parcellation, the results were more complicated. Not only did the models allowing error variances to differ over timepoints outperform the standard ICED constrained models across most regions. We also found serious issues with model fit using the Destrieux parcellation, suggesting that alternative methods to estimate reliability and longitudinal stability may be needed as well as further investigation into the role of image processing and parcellation on the longitudinal stability across brain regions.

### 4.1 Practical Implications

Our results have several implications. First, we should expect associations between cortical thickness and a phenotypic variable to be more attenuated on average than associations between surface area or volume and the same phenotypic variable. We demonstrated that for cortical thickness, three or more repeated measures would be needed for most brain regions to achieve high longitudinal stability to ensure true associations are not overly attenuated. We also highlighted that differences in longitudinal stability between regions can lead to requiring as many as 70% more participants to achieve the same level of statistical power. Of course, the relationship is nuanced, depending on the particular region of interest, the “true” association of interest, and other characteristics of the model and sample. For instance, in very underpowered studies, we are just as likely to see attenuation as over-estimation of our effects, also known as Type M (magnitude) errors (Gelman & Carlin, 2014). This effectively increases the chances of false-positive effects observed in small sample studies, or studies with too few repeated measure studies, further exacerbated in the case of significant threshold-driven publication bias (Loken & Gelman, 2017).

Second, our results highlight the challenge inherent to comparing relative contributions of brain regions and structures without assessing the measurement properties across measures and regions. Alongside our results (Section 3: practical implications), we include practical examples of differential effect size attenuation resulting from differences in longitudinal stability across brain regions. If these spatial differences (or, indeed, measure differences) are systematic across samples, then our empirical associations will be affected by these patterns, regardless of the true pattern of associations. Thus, systematic patterns of longitudinal stability may hide or even induce patterns of spatial specificity. This is especially important when studying populations where processes of key interest (as is the case for ABCD; Casey et al., 2018; B. J. Casey, Getz, & Galvan, 2008; B. J. Casey, Jones, & Hare, 2008; Steinberg, 2008) such as functional and structural changes in the (pre)frontal lobes and their associations with (changes in) risk-taking behaviour are predominantly focused on regions that may, for methodological reasons, have undesirably low reliability. Estimating and reporting longitudinal stability and stability as standard practice (c.f. Parsons et al., 2019) affords us the opportunity to correct our estimates (e.g., Cooper et al., 2017; Schmidt & Hunter, 1996), or use approaches that integrate longitudinal stability into the model (e.g., for cognitive measures, see Haines et al., 2020; Rouder & Haaf, 2018). Both would facilitate comparisons across elements where we know longitudinal stability and stability likely differ (region, measure, sample, etc).

We stress that the implications of these analyses stretch further than grey matter measures in the ABCD data. Although the precise longitudinal stability estimates of these metrics will likely vary in other samples as a function of the nature of the study design, participant demographics, scanner specification, and other aspects, we believe several of our high-level findings are likely to generalize. First and foremost, reliability and longitudinal stability are likely to vary across brain regions, measures, and samples. For example, MRI and fMRI show distinct patterns of longitudinal stability (Elliott et al., 2020), shorter term reliability has also been shown to differ across channels in functional near infrared spectroscopy (Blasi et al., 2014) and EEG components (McEvoy et al., 2000). Beyond brain measures and regions, it has been demonstrated that different fMRI data processing pipelines can lead to marked variation in results, even using the same data (Li et al., 2021). Similarly, in behavioural data, even basic data-cleaning decisions can lead to large variation in reliability and longitudinal stability (Parsons, 2022).

In sum, we may find different patterns of longitudinal stability across: imaging modalities (e.g., EEG, NIRS), analyses pipelines, brain regions and parcellations, populations and studies, as well as over the lifespan. We argue that reliability (and longitudinal stability) varies across a number of factors, and the unrevealed variation in reliability poses a danger to our inferences. Much more work is needed to ensure we understand the psychometric properties of our tools, and the heterogeneity of these properties across modalities. In future studies, ICED models (Brandmaier, Wenger, et al., 2018) could be expanded with moderation approaches (Bauer, 2017) to directly examine predictors of error, such as time between scans, head movement, testing site and researcher-related differences, and demographic characteristics. By systematically accounting for these between-site and between-subjects features, we can further improve longitudinal stability and stability estimates, while investigating how researchers could minimise these sources of error in future study designs.

### 4.2 Limitations and opportunities for future research

The central limitation of this paper is the reliance on two-timepoint data. Currently, ABCD (Casey et al., 2018) has collected and released access to two timepoints of imaging data (with an average of 2 years between scans). As such, we did not examine sources of variance that could be possible in more complex testing schemes with three or more timepoints (e.g. Anand et al., 2022; Brandmaier, Wenger, et al., 2018; Wenger et al., 2021) Prior work has examined the within-session reliability of fMRI measures within ABCD (Kennedy et al., 2022). However, to our knowledge, ABCD did not collect similar within-session repeated structural measures—we therefore focused on longitudinal stability. Future investigations would benefit from including repeated measures within session to enable the teasing apart of reliability and longitudinal stability and allow us to investigate predictors of both (For an example of using the Generalizability Theory, see Noble et al., 2017). In this paper, we chose instead to capitalise on the multi-site nature of ABCD to examine sources of variance across brain regions and testing sites.

As we highlight in the introduction, individual differences in rate of change in brain structure over time will reduce our stability estimates. With two timepoints, we cannot uniquely identify these individual differences in change. As such, while high stability suggests we can adequately rank-order participants over this time period, it does not suggest that participants’ brain structure remained stable across that time period (e.g., if all participants’ cortical thickness increased by 1 mm, the stability estimates would be identical here). On the other hand, low stability indicates that we are unable to adequately rank-order participants in this time course. This could result from population-level instability; it could suggest that the rate of change between participants differs substantially. However, these estimates alone do not give us information about the other sources of within-subject variance. Lifespan charts of brain development (Bethlehem et al., 2022) highlight periods of rapid change and stability in brain structure, as well as periods characterised by greater between-subjects variance. Moving forward, developmental neuroscience needs models that capture the reliability of change, alongside a sufficient number of repeated measures (longitudinal and ideally within session). We suggest two ways this might be achieved with extensions of the ICED modelling approach.

First, to model change in two timepoint data, many studies calculate change scores (or annualised change scores to account for differential timings between scans). Difference scores can be modelled equivalently within the SEM framework as latent difference scores (for a tutorial, see Kievit et al., 2018). It is possible to extract reliability estimates for these change scores, with some adaptations to the ICED approach (the difference score model is a special case of a two-timepoint latent growth curve model). It is worth noting that the literature on the reliability of difference scores indicates we should expect generally lower reliability than individual measures (e.g. Lord, 1956; Thomas & Zumbo, 2012; Zimmerman & Williams, 1998). Unfortunately, in standard latent difference score models, the error variance is not uniquely identifies; instead, they only capture the variance of the intercept and the change, which are both confounded with error in a single-indicator model. In effect, the model specification assumes perfect reliability of the change score if the intercept and change are to be interpreted as “pure” constructs. Given an estimate of reliability, or multiple indicators at each timepoint (e.g., multiple scans per session), the reliability of the change score can be estimated. Further work in this direction would enable mapping the reliability of change, given only two timepoints, as we have done in this paper across measures and brain regions.

Second, with three or more timepoints (e.g., when further waves of ABCD data are released), the ICED models can be expanded into latent growth curve models (Brandmaier, von Oertzen, et al., 2018; Brandmaier, Wenger, et al., 2018). This powerful and flexible extension allows for the simultaneous modelling of the intercept and slope reliability, termed “Effective Curve Reliability.” This approach would provide key insights for investigations of individual differences in trajectories of change in existing and future data. Further, using this approach, we can directly incorporate the non-linear changes in brain structure known to occur throughout the lifespan (e.g. Bethlehem et al., 2022). Psychometrically, this would allow us to expand the grey matter structure reliability maps presented in this paper into reliability maps of change trajectories allowing us to gauge how well we can detect individual differences, their antecedents, correlates, and consequences. Effective curve reliability is a valuable tool for planning future studies for desired levels of precision, expected reliability, and statistical power, given variance estimates from studies such as ours and the planned longitudinal sampling. These considerations become especially important in clinical applications, such as drug trials intending to decelerate atrophy in MS or dementia, given the time and expense required to conduct longitudinal neuroscience.

Our additional exploratory analyses raises several additional limitations and avenues for future investigations. The pattern of longitudinal stability we observed using the Destrieux parcellation (mean = .20, range = .00 to .50) was markedly poorer than using the Desikan-Killiany-Tourville parcellation (mean = .76, range = .54 to .90; also see Fig. 11). However, Freesurfer version 6.0 was used for the Destrieux parcellation analysis, while version 5.3 was used for the ABCD data release. Several major improvements were made between versions, including moving from calculating grey matter volume as the product of surface area and cortical thickness (Freesurfer 5.3 – used in the ABCD release) to an irregular polyhedron approach (Winkler et al., 2018 - used from Freesurfer version 6.0 and for the Destrieux parcellation analyses). We suggest that further investigations of the impact of processing pipelines and software versions on reliability and longitudinal stability are warranted (e.g., see Li et al., 2021). This could further extend to assessing the progress of new software versions against older ones. Further, we note that the Destrieux parcellation analysis used a different data processing pipeline (Rutherford et al., 2022) compared to the ABCD data releases (Hagler et al., 2019), and the Destrieux parcellation analysis used a subsample of the full ABCD sample. In sum, our results demonstrate that cortical parcellations are impactful on longitudinal stability. Researchers will need to consider the trade-off between anatomical fidelity and stability (or reliability), depending on the goals of the study. An exciting line of future research will be to characterise parcellation-related differences in longitudinal stability and reliability—including parcellations we did not use here, for example, the Glasser parcellation (Glasser et al., 2016) and the human Brainnetome atlas (Fan et al., 2016)

### 4.3 Summary

In this study, we mapped the (2-year) test-retest stability of grey matter measures across brain regions using the first two timepoints from the ABCD study (Casey et al., 2018). This study complements previous examinations of the reliability and longitudinal stability of fMRI measures (Kennedy et al., 2022; Taylor et al., 2020). It also adds to existing research on the test-retest reliability and longitudinal stability of structural MRI measures (Elliott et al., 2020; Han et al., 2006), focusing on a longer timescale. Previous studies have used relatively short inter-scan intervals, for example, 2 weeks: we moved beyond prior investigations and examined longitudinal stability in a very large sample with 21 testing sites across a longer developmental period (2 years). We found patterns of stability to differ across structural measures, brain regions, and testing sites. Decomposing these estimates allowed us to highlight that differences in stability across brain regions appear to be largely due to genuine between-subjects differences. In contrast, differences in stability across testing sites were driven by variations in error, hinting at important cross-site differences causing increases in measurement error. Heterogeneity in reliability or longitudinal stability is not a problem in itself, but it does highlight the importance of examining the reliability of our measurements, and further investigating the sources of this (un)reliability, or longitudinal (in)stability, variance. We offered suggestions for expanding the Intra-Class Effect Decomposition approach used here into future investigations. Further detailed mapping of the reliability and longitudinal stability of structural brain measures over the lifespan should facilitate improving the efficiency and accuracy of developmental cognitive neuroscience.

## Data Availability

We used imaging data from the Adolescent Brain Cognitive Development study (Casey et al., 2018), data release 4.0 (http://dx.doi.org/10.15154/1523041). Data are available upon request and approval from NIMH Data Archive (NDA). The code used for these analyses can be found in the OSF (https://osf.io/rxmn2/; timestamped registration https://osf.io/ukjvm) and the github repositories for this project (https://github.com/sdparsons/Longitudinal_Stability_ABCD_Grey_Matter). Data from ABCD may be obtained via application from the NIMH Data Archive (https://nda.nih.gov/abcd/). The ABCD data used in this report came from release 4.0 (http://dx.doi.org/10.15154/1523041; accessed on 21st February 2022). Readers may be interested in applying these methods to their own data or in reproducing our analyses. To make these analyses accessible and to make the *ICED* package easy to use, we simulated data for each structural measure based on the ICED variance estimates (separately for each testing site and matching the sample size at each site) and provided these in the Supplementary Materials.

## Footnotes

1 Note: unless reliability is explicitly specified in the model, most statistical tools assume perfect reliability.

2 There have been several recommendations for standards to judge test-retest reliability (here, longitudinal stability) estimates. For example, a common historical rule of thumb is < 0.4 is poor, 0.4 – 0.59 is fair, 0.6 – 0.74 is good, and 0.75 – 1 is excellent (Cicchetti & Sparrow, 1981; Fleiss, 1986). Others have proposed stricter rules of thumb of < 0.5 is poor, 0.5 – 0.75 is moderate, 0.75 – 0.9 is good, and 0.9 – 1 is excellent (Koo & Li, 2016). In this paper we avoid adopting any specific threshold to describe our reliability estimates. We aim to avoid dichotomous thinking about whether estimates are ‘good’ or ‘bad’ in favour of considering the influence of relative differences in reliability across different measures, regions, and sites.

3 We also present the AIC and BIC model comparisons in the supplemental materials.

4 We would like to thank an anonymous reviewer for highlighting this key point.

5 Sesame Street, Episode 1056 (1977).

## Author Note

We would like to thank Léa Michel for sharing code to map ABCD grey matter measures to the ggseg package for visualisation. We would also like to thank André Marquand for sharing data processed using the Destrieux parcellation; this allowed us to complete our additional exploratory analyses.

## Supplementary Materials

The code used for these analyses can be found in the OSF (https://osf.io/rxmn2/; timestamped registration https://osf.io/ukjvm) and the github repositories for this project (https://github.com/sdparsons/Longitudinal_Stability_ABCD_Grey_Matter).
